# Nosocomial Malaria and Saline Flush

**DOI:** 10.3201/eid1107.050092

**Published:** 2005-07

**Authors:** Sanjay K. Jain, Deborah Persaud, Trish M. Perl, Margaret A. Pass, Kathleen M. Murphy, John M. Pisciotta, Peter F. Scholl, James F. Casella, David J. Sullivan

**Affiliations:** * Johns Hopkins University, Baltimore, Maryland, USA

**Keywords:** molecular epidemiology, infection, case report, Malaria, nosocomial, microsatellite, plasmodium falciparum, transmission, blood-borne, mass spectrometric

## Abstract

An investigation of malaria in a US patient without recent travel established *Plasmodium falciparum* molecular genotype identity in 2 patients who shared a hospital room. *P. falciparum* can be transmitted in a hospital environment from patient to patient by blood inoculum if standard precautions are breached.

Almost all of the 1,400 cases of malaria reported each year in the United States are acquired by mosquito bite during travel in malaria-endemic areas (1). However, mosquito transmission in the United States accounts for a few cases each year (2). Nosocomial malaria represents person-to-person transmission of parasite-infected erythrocytes through blood transfusion, needlestick injury, improper use of blood glucometers, multidose heparin vials, organ transplantation, contaminated catheters that deliver contrast medium, or rarely, open wounds (3–6).

Nosocomial transmission of malaria secondary to inpatient nursing practices has never been reported in the United States. We report nosocomial transmission of *Plasmodium falciparum*, confirmed by molecular genotyping, by improper use of saline flush syringes in a tertiary care hospital in the United States.

## The Study

Abdominal pain, emesis, and a high fever developed in patient 1, a 9-year-old Gambian boy with sickle cell disease residing in the United States, during the flight home after a month in the Gambia; he had taken no malaria prophylaxis drugs. After diagnosis of *P. falciparum* malaria with 4% parasitemia and transfer to unit A of a tertiary care hospital, he responded well to antimalarial therapy and was discharged 2 days later.

Seven days before patient 1's admission, patient 2, a 14-year-old girl, with severe developmental delay, was admitted to unit A for placement of a surgical feeding tube. Patients 1 and 2 shared a unit A semiprivate room for ≈24 hours. Patient 1 received a continuous quinidine gluconate infusion through a peripheral intravenous line. Chart review and interviews indicated that neither patient had glucose monitoring by glucometer, blood transfusions, common infusions such as contrast material, or a needlestick injury report, all events that have been previously implicated in nosocomial malaria. Medicine doses given and blood samples drawn were documented with a separation time >50 minutes. A week after discharge and 17 days after sharing the semiprivate room, patient 2 was admitted to another hospital (3-day stay), and a viral illness was diagnosed after all cultures were negative for pathogens. Persistent fever and pancytopenia developed and resulted in her readmission to unit A of the tertitary care hospital, now 23 days after sharing the room with patient 1. Patient 2 was febrile and pale, with no notable change in her baseline neurologic state. The leukocyte count was 1,890 cells/mm^3^, hematocrit was 35.2%, platelets were 47,000/mm^3^, and total bilirubin was 1.5 mg/dL. Despite administration of antimicrobial drugs for presumed microbial sepsis, the following conditions developed over the next 72 hours: persistent spiking fevers; loose, nonbloody stools; splenomegaly; abdominal distension; and bilateral lower extremity edema. Formal review of her peripheral blood smear by the hematology consultation service showed intraerythrocytic ring forms indicative of *P. falciparum* malaria, with 12% parasitemia and ≈200 gametocytes/mm^3^. After 3 blood transfusions and quinidine gluconate and doxycycline therapy, the patient was discharged on hospital day 14, with a stable hematocrit, persistent gametocytemia, and splenomegaly. Initial and follow up tests on patient 2 were negative for viral bloodborne pathogens. The patient lives with her parents in the Baltimore-Washington area ≈12 miles from the nearest international airport. She had no history of recent travel to any areas that are endemic for malaria. No locally acquired cases of malaria had been reported in the regional area. This prompted an investigation to look for a nosocomial route of transmission. Approval for the study was obtained from Johns Hopkins University

Two potential sources of nosocomial transmission in unit A were identified: 1) heparin syringes, which were filled from multidose vials, and 2) factory preloaded 10-mL saline flushes, of which up to 3 mL was used per flush for intravenous lines not in use. Hospital policy had strict guidelines for using multidose devices and did not allow reusing single use devices. However, interviews of 7 nurses, including those who cared for the 2 patients, showed that 2 nurses admitted reusing saline flushes on the same patient, and 4 nurses had observed saline flushes being reused in unit A. Reusing multidose heparin vials was not documented.

**Figure 1:**
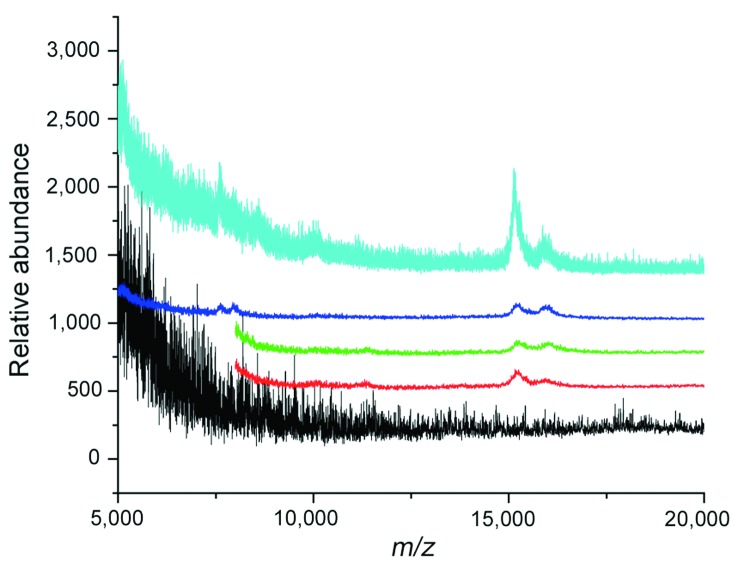
Mass spectroscopic analysis of sterile saline flush syringes after routine use. The contents of the used syringes were concentrated by centrifugation. Matrix-assisted laser desorption ionization detected the α and β chains of hemoglobin as the ions at mass/charge (*m/z*) 15,126 and 15,867, respectively, in samples A (red), B (green), C (blue), and J (aqua) that were absent in the matrix alone (black). The lower limit of sensitivity with matrix-assisted laser desorption ionization is ≈0.5 erythrocytes per mL.

To determine whether used saline flush syringes that were visibly clear contained blood, we tested saline from 8 used syringes and 2 unused controls. The contents of only 1 used saline flush syringe visibly contained blood. After the contents were centrifuged to concentrate erythrocytes, blood was found in 4 of 8 of the used syringes (Figure 1) by using matrix-assisted laser desorption ionization/time-of-flight mass spectrometry (7). No blood was found in the unused syringes.

**Figure 2:**
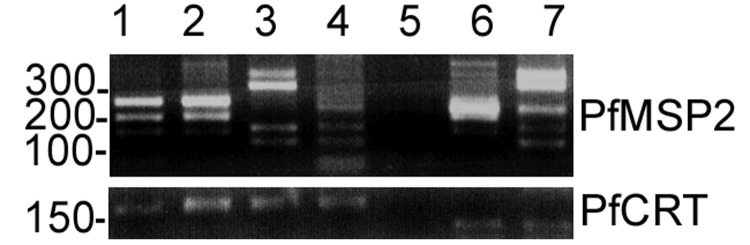
Genotype analysis of patient blood films. Restriction fragment length polymorphism are shown for *Pfmsp2* and *Pfcrt*. Patient 1 and 2 are identical at the polymorphic *Pfmsp2* (lanes 1 and 2), while unrelated patient controls (lanes 3 and 4) are different. One of the 2 negative controls is shown (lane 5). Genomic DNA from clone HB3 (lane 6) and isolate NF54 (lane 7) are also included as additional positive controls. All recent patient samples have the *Plasmodium falciparum* chloroquine-resistant genotype.

Polymerase chain reaction (PCR) amplification of *P*. *falciparum* genomic DNA, isolated with DNAzol (Invitrogen, Carlsbad, CA, USA), was successful from the archived Giemsa blood films from both patients and 2 other patients (not related in time or travel history) infected with *P*. *falciparum* malaria seen at our institution in the past year. Figure 2 shows that the PCR product restriction digests for the polymorphic *P. falciparum* merozoite protein 2 (*Pfmsp2*) (8) and the *P. falciparum* chloroquine resistance transporter (*Pfcrt*) (9) gene fragments were identical for patients 1 and 2. This identity was also confirmed by sequence analysis.

**Figure A1:**
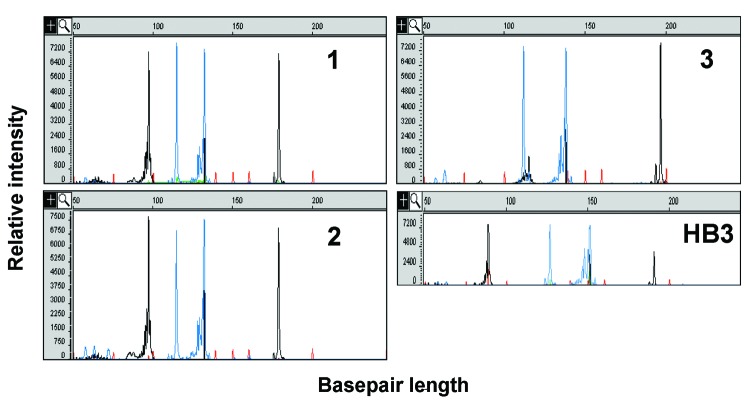
Capillary electropherograms of *Plasmodium* microsatellite analysis. The x axis represents fragment size in bases and the y axis represents fluorescence intensity. The *P. falciparum* microsatellites products C13M30 and PFPK (black peaks) and TA81 and C4M8 (blue peaks) from amplification genomic DNA from patients 1, 2, one of the unrelated patient controls, and control *P. falciparum* clone HB3. Each microsatellite has ≥10 alleles. Patients 1 and 2 have identical microsatellites at all 4 loci (Table).

We also examined 4 *P*. *falciparum* microsatellite alleles (TA81, PFPK, C13M30, C4M8) at independent chromosomal loci by a fluorescent-tagged heminested PCR amplification (10). Patients 1 and 2 had identical *Plasmodium* microsatellite lengths at all 4 loci; each of the positive controls was different as shown in Figure 2, the Figure A1, and the Table. With the simplistic assumption of ≥10 alleles at each microsatellite loci and *Pfmsp2*, the chance occurrence of identity is <1:100,000. By using the AmpFLSTR Profiler kit (Applied Biosystems, Foster City, CA, USA), subsequent forensic analysis of the human genomic DNA from leukocytes in the same isolated *P*. *falciparum* DNA samples from the blood films demonstrated the sex difference between patients 1 and 2. The analysis also demonstrated the differences at 9 human microsatellite loci, which indicated that cross-contamination during processing was not a likely source for the similarities detected.

**Table:** Plasmodium falciparum microsatellite lengths in number of base pairs

Patient#	TA81	PFPK	C13M30	C4M8
1	114	178	97	132
2	114	178	97	132
3	111	196	115	138
4	126	178	97	154
HB3	126	190	89	151

## Conclusions

Natural transmission of malaria between patients 1 and 2 by a mosquito is not possible within 24 hours; the maturation cycle from ingestion of viable gametocytes in a blood meal to infective sporozoite stage in the mosquito salivary glands is 8–35 days (11). Direct mechanical transmission of parasitized blood by an arthropod vector is a theoretical possibility, but no such transmission has ever been reported, and the female *Anopheles* mosquito does not regurgitate blood upon refeeding. Another remote possibility, luggage malaria, is the introduction of an already infectious mosquito from the Gambia brought into unit A through patient 1's luggage (12). If this were the case, a different parasitic genotype would be expected in patients 1 and 2 because the wild mosquito that infected patient 1, from 1 to 2 weeks before onset of symptoms, would have died 2–6 days after delivering the infective bite. Likewise, an asymptomatic traveling companion of patient 1 would have a distinct parasite genotype.

In this report, epidemiologic investigation excluded many nosocomial routes of transmission. Potential sources not excluded were multidose heparin vials and saline flush syringes. However, identifying reuse of saline flush syringes in the unit and detecting blood in clear, used saline flush syringes suggested that this was the most likely source. Finally, molecular identity of *P*. *falciparum* isolated from patients 1 and 2, as assessed by restriction fragment length polymorphism of *Pfmsp2*, and forensic microsatellite analysis of *P. falciparum* genotypes confirmed that contaminated fluid from patient 1 was the source of the infection in patient 2. Together, these data strongly suggest that apparently clear solution in a used saline flush syringe containing infected *P. falciparum* erythrocytes, was reused on a neighboring patient, leading to nosocomial transmission of malaria. Saline flush vials and bags have been implicated in nosocomial transmission of hepatitis B and microbes, respectively (13,14).

Theoretically, a single infected erythrocyte is able to transmit malaria by inoculation. Patient 1 had almost 200 million infected erythrocytes per milliliter of blood based on parasitemia level. Based on our data, apparently clear saline can contain ≈1 million erythrocytes per milliliter. Therefore a single microliter of patient 1's blood diluted into 5 mL of saline would be almost 40,000 infected erythrocytes per milliliter of an apparently clear solution.

Our report illustrates that nosocomial transmission can occur in a tertiary care setting in the United States, despite healthcare workers' access to gloves, disposable needles, intravenous devices, and flushes. The common perception that flushing practices are not associated with the aspiration of minute amounts of blood contributed to this occurrence of nosocomial malaria. Our institution, like many others, has used such episodes to facilitate change and refocus on patient safety. The interventions initiated specifically included retraining of pediatric staff about 1) no reuse of single use saline flushes, 2) transmission of bloodborne pathogens with an emphasis on nonviral pathogens, and 3) removal of multidose vials, including those with heparin, from pediatric units. Warnings about the potential risks of reusing saline flush syringes have been incorporated into institution-wide safety training. Ongoing rigorous review of healthcare practices and strict adherence to body and fluid precautions are essential for minimizing patient exposure to highly infectious pathogens, even in resource-rich settings.

## Acknowledgments

We thank Lirong Shi, Karen Mackie, and the pediatric intensive care unit and hospital epidemiology and infection control staff who assisted in this investigation. We also thank Stephen Dumler for his continued encouragement and support.

The AB-Mass Spectrometry Facility at the Johns Hopkins School of Medicine is funded by National Center for Research Resources Shared-Instrument Grant 1S10-RR14702. NCCR grant GPDGCRC RR0052 supported the culture of *P. falciparum* for control DNA.
